# Evaluation of circulating cell-free DNA in cholestatic liver disease using liver-specific methylation markers

**DOI:** 10.1186/s12876-021-01741-5

**Published:** 2021-04-01

**Authors:** Sohan Punia, Brian D. Juran, Ahmad H. Ali, Erik M. Schlicht, Raymond M. Moore, Zhifu Sun, Konstantinos N. Lazaridis

**Affiliations:** 1 Division of Gastroenterology and Hepatology, College of Medicine, Mayo Clinic, 200 First Street SW, Rochester, MN 55905 USA; 2 Division of Biomedical Statistics and Informatics, Mayo Clinic, Rochester, MN 55905 USA

**Keywords:** PBC, PSC, CfDNA, Liver, Cholestasis

## Abstract

**Background:**

Quantification of circulating organ-specific cell-free DNA (cfDNA) provides a sensitive measure of ongoing cell death that could benefit evaluation of the cholestatic liver diseases primary biliary cholangitis (PBC) and primary sclerosing cholangitis (PSC), which lack reliable non-invasive biomarkers. Our goal in this pilot study was to determine whether liver-specific cfDNA levels are increased in PBC and PSC patients relative to controls and in advanced versus early disease, to evaluate their potential as novel disease biomarkers.

**Methods:**

Peripheral blood derived bisulfite-treated DNA was PCR amplified from patients with PBC (n = 48), PSC (n = 48) and controls (n = 96) to evaluate methylation status at 16 CpG sites reported to be specifically unmethylated in liver tissue near the genes IGF2R, ITIH4 and VTN. Amplicons were used to prepare paired end libraries which were sequenced on a MiSeq sequencer. Trimmed reads were aligned and used to determine unmethylation ratios and to calculate concentration of liver-specific cfDNA. Comparisons between groups were performed using the two-tailed Mann–Whitney Test and relationships between variables were evaluated using Pearson’s Correlation.

**Results:**

Levels of liver-specific cfDNA, as measured at the 3 genetic loci, were increased in PBC and PSC patients relative to controls and in late-stage relative to early-stage patients. As well, cfDNA levels were correlated with levels of alkaline phosphatase, a commonly used biochemical test to evaluate disease severity in liver disease, in patients, but not in controls.

**Conclusions:**

cfDNA offers promise as a non-invasive liquid-biopsy to evaluate liver-specific cell-death in patients with cholestatic liver diseases.

## Background

Primary biliary cholangitis (PBC) and primary sclerosing cholangitis (PBC) are rare, cholestatic liver diseases of unknown etiology. Disease pathogenesis in PBC and PSC is complex, involving genetic and environmental factors, and despite both being immune-mediated cholestatic liver diseases, there are significant differences. For instance, PSC involves inflammation and fibrosis in both the intra- and extra-hepatic bile ducts, while in PBC only the intra-hepatic ducts are affected [1]. PSC primarily affects males (~ 60%) while PBC affects mainly females (~ 90%) [1]. The majority of PSC patients (~ 80%) have co-existing inflammatory bowel disease (IBD), whereas PBC patients only rarely have IBD [1]. Similarly, PSC patients have a highly elevated risk of liver and colon cancers while PBC patients do not [1]. Finally, there are approved medications to treat PBC, ursodeoxycholic acid and obeticholic acid, neither of which has FDA approval for use in PSC, which currently lacks therapeutic options [2]. Regardless of the differences, both PBC and PSC are progressive diseases and orthotopic liver transplantation (OLT) is eventually required in many patients [3, 4]. Despite some recent progress, PSC and PBC still lack reliable non-invasive prognostic biomarkers [5], hampering the prediction of disease outcomes and assessment of the effect of therapy [6]. To address this unmet need, we have utilized an assay designed to detect liver-specific circulating cell-free DNA (cfDNA) in plasma as a potential prognostic biomarker for PBC and PSC.

Apoptotic and injured dying cells are constantly releasing DNA into the blood and levels of this cfDNA have been shown to increase in cancer, cardiovascular disease, sepsis, autoimmune diseases and following intensive exercise [7–11]. Detection of cfDNA coming from particular organs relies on DNA methylation signatures that are organ specific. Such signatures have recently been reported for a wide range of tissues and cell types including the liver [12–14]. For instance, a recent study reported CpGs near the genes IGF2R, VTN and ITIH4 to be specifically unmethylated in the liver and showed these marks to be detectable in plasma of normal controls and increased following liver transplantation and in the context of liver damage in the setting of sepsis [12]. However, other liver pathologies were not assessed in this report. Our goal in this pilot study was to determine whether the levels of these liver-specific unmethylated CpGs are increased in the plasma cfDNA of PBC and PSC patients relative to controls and in late-stage versus early-stage disease, as a means to evaluate their potential utility as novel disease biomarkers.

## Methods

### Study subjects

The study was approved by the Mayo Clinic Institutional Review Board and conforms to standards laid out in the Declaration of Helsinki. All participants provided written informed consent. Patients with PSC were selected from the PSC Resource of Genetic Risk, Environment and Synergy Studies (PROGRESS) [15] and patients with PBC were participants of the Mayo Clinic PBC Genetic Epidemiology Registry and Biospecimen repository [16]. As age and sex distributions differ between PBC and PSC, separate control populations with no history of liver disease were selected for each disease from the aforementioned resources. The diagnosis of PSC and PBC was based on standard clinical, biochemical, cholangiographic and histological criteria [17, 18]. PBC and PSC patients were selected to equally represent early and late disease stages. For PSC, late disease was defined as having serum alkaline phosphatase (ALP) greater than 3 times the upper limit of normal (ULN) and/or bilirubin greater than 2.5 mg/dL at time of sample collection or progression to OLT within 4 years of follow up. Late PBC was defined similarly, although bilirubin values were not available. Early PSC and PBC was defined as having ALP less than 1.1 times the ULN at sample collection with no evidence of elevated bilirubin, cirrhosis or OLT in follow up.

### Plasma and cfDNA preparation

Plasma samples were collected in EDTA-containing tubes and stored at − 80 °C prior to use. Thawed samples were centrifuged two times for 10 min at 1500 rpm at 4 °C to remove cellular debris and the supernatant was stored at –80 °C prior to further processing. cfDNA was extracted from 2 ml of plasma using the Qiagen Cell-Free DNA (cfDNA) Purification Kit (Qiagen) and cfDNA concentration was measured using Qubit (Thermo Scientific). The cfDNA was then treated with bisulfite using the Zymo Research- EZ DNA Methylation-Gold™ Kit (Zymo Research) following the manufactures recommended protocol.

### Next generation sequencing

Bisulfite-treated DNA was PCR (multiplex) amplified using the Qiagen multiplex PCR kit (Qiagen) using primers specific for bisulfite-treated DNA but independent of methylation status at 16 monitored CpG sites in the vicinity of IGF2R (6 CpGs), VTN (5 CpGs) and ITIH4 (4 CpGs), which are specifically unmethylated in liver tissue, as described previously [12]. Primer sequences were, IGF2R: L: TGGGTGTTGTTATTTTGTTGA and R: CTACAAAAATACACACCCCAA (94 bp); ITIH4: L: ATAGTGAAGATGTTAGTTTGTTTTT and R: AACACACTTACCTAATAACCAAAC (137 bp); VTN: L: GGTATTTTGAAGAGGTAGGTTT and R: ACCTAAATACCCCAAACTCAT (108 bp) and CpG locations are provided in Table 1. PCR products were cleaned with ExoSap-IT (Thermo Scientific) and sent to the genome analysis core at Mayo Clinic for library preparation and sequencing. Quality and quantity of amplicon DNA were analyzed by Qubit (Thermo Scientific) and bioanalyzer (Agilent). Individual paired end libraries were prepared using the NEBUltra II kit (New England Biolabs) without DNA fragmentation. As the combined read length of the 3 multiplex amplicons were only 339 bp, each disease/control group of 96 samples were barcoded and sequenced on a single lane of a MiSeq sequencer (Illumina).

**Table 1:** CpGs targeted in the NGS assay

CpG name	Chromosome	Location^a^	Read count median (IQR)	Unmethylated ratio median (IQR)
ITIH4-1	3	52,830,996	36,680 (31,875–44,483)	0.280 (0.216–0.346)
ITIH4-2	3	52,831,009	36,718 (31,854–44,520)	0.035 (0.012–0.067)
ITIH4-3	3	52,831,051	36,624 (31,843–44,426)	0.089 (0.064–0.129)
ITIH4-4	3	52,831,059	36,420 (31,637–44,149)	0.099 (0.075–0.137)
IGF2R-1	6	160,079,556	38,527 (31,251–44,719)	0.049 (0.025–0.077)
IGF2R-2	6	160,079,562	38,498 (31,238–44,699)	0.041 (0.023–0.066)
IGF2R-3	6	160,079,586	38,584 (31,288–44,758)	0.033 (0.015–0.062)
IGF2R-4	6	160,079,588	38,574 (31,292–44,757)	0.038 (0.019–0.066)
IGF2R-5	6	160,079,593	38,564 (31,255–44,715)	0.030 (0.015–0.056)
IGF2R-6	6	160,079,595	37,314 (30,258–43,314)	0.035 (0.020–0.069)
IGF2R-7	6	160,079,606	36,947 (30,090–43,003)	0.033 (0.021–0.067)
VTN-1	17	28,369,323	10,402 (8209–13,100)	0.064 (0.030–0.108)
VTN-2	17	28,369,333	10,365 (8187–13,077)	0.045 (0.014–0.086)
VTN-3	17	28,369,339	10,646 (8210–13,097)	0.049 (0.019–0.095)
VTN-4	17	28,369,351	10,379 (8195–13,060)	0.052 (0.023–0.092)
VTN-5	17	28,369,368	9058 (6937–11,259)	0.062 (0.030–0.109)

^**a**^HG38 human genome coordinates

### Bioinformatics and statistical data analysis

Adapter sequences were trimmed from the de-multiplexed raw sequence data in fastq format using Trim Galore [Trim Galore v0.4.4, https://www.bioinformatics.babraham.ac.uk/projects/trim_galore/]. Paired-end reads greater than 20 bases long after trimming and low quality base removal were aligned to human reference genome hg38 using BSMAP (v2.73) [19] with default parameters, followed by sorting and indexing the aligned BAM files. Methylation data was extracted for uniquely mapped read pairs from aligned bam files by a BSMAP script and the data was merged by CpG position across all samples. Off-target CpG sites were excluded and only the 16 targeted CpGs were analyzed further. CpGs were considered unmethylated if “TG” was read and methylated if “CG” was read. We determined absolute levels of cfDNA in genome equivalents per ml (Geq/ml) as previously described [14]. Briefly, we calculated the unmethylation ratio for each locus by dividing the number of unmethylated reads by the total number of reads for all included CpGs. Then, we multiplied this ratio by the total concentration of cfDNA isolated from the 2 ml plasma sample. Finally, we converted from units of ng/ml to genomic equivalents per ml by multiplying by a factor of 303, assuming the mass of a single haploid genome to be 3.3 picograms. The values obtained represent the amount of liver-specific cfDNA in circulation, as measured for each locus, and were used in downstream analyses. Categorical variables were compared using chi-square or Fisher’s exact test and continuous variables were compared using the Mann–Whitney test whereby values were expressed as median and inter-quartile range (IQR). Correlation between variables was determined by calculating the Pearson correlation coefficient. P-values of 0.05 or less were considered significant.

## Results

### Patient characteristics

A total of 48 PBC patients and 48 PSC patients were selected and matched to separate groups of 48 unaffected controls based on sex, reported race and age at sample collection. Following data generation, one of the PSC patients was found to be an outlier, having liver-specific DNA levels greater than twofold higher at each locus than all other patients, and was removed from the study, leaving 47 PSC patients. The characteristics of these patient-control groups are presented in Table 2 (PBC) and Table 3 (PSC). The patient groups were further separated into two groups of 24 patients with early- or late-stage disease based on biochemical and clinical data. These groups were well-matched for most parameters, but PBC patients with late-stage disease were younger at diagnosis than those with early disease, median 42.9 years versus 52.1 years, respectively, *p* = 0.0185 (Table 2). In PSC this trend was opposite, with advanced disease patients being diagnosed later than patients in the early disease group, median 46.1 years versus 36.2 years, respectively. However, this difference was not statistically significant, *p* = 0.0693 (Table 3).

**Table 2:** Characteristics of PBC patients and controls

	Controls (n = 48)	PBC (all) (n = 48)	*p*-value^a^	PBC (early) (n = 24)	PBC (late) (n = 24)	*p*-value^b^
Sex, % male	12.5	12.5	> 0.9999	8.3	16.7	0.6662
Race, % Caucasian	98	91.7	0.3616	95.8	87.5	0.6085
Age at sample collection (yrs), median (IQR)	56.3 (48.2–61.0)	56.3 (41.3–56.5)	0.9927	57.8 (53.3–62.2)	51.7 (46.5–61.0)	0.1071
ALP (xULN), median (IQR)	0.60 (0.48–0.73)	1.17 (0.83–4.02)	< 0.0001	0.83 (0.71–0.94)	3.98 (3.17–5.33)	< 0.0001
AMA, (% positive)	0	81.3	< 0.0001	79.2	83.3	> 0.9999
Age at Dx (yrs), median (IQR)	na	49.4 (41.3–56.5)	–	52.1 (46.3–57.4)	42.9 (39.9–51.9)	0.0185
Disease duration (yrs), median (IQR)	na	5.0 (2.0–9.0)	–	4.5 (2.0–9.0)	5.5 (2.0–10.8)	0.6116
Clin. FU (yrs), median (IQR)	na	6.0 (4.0–10.0)	–	8.5 (5.3–10.0)	5.0 (2.0–8.8)	0.0133
UDCA treatment, (%)	na	97.9	–	100	95.8	> 0.9999

^**a**^*p*-value for control versus PBC (all) comparison

^b^*p*-value for PBC (early) versus PBC (late) comparison; ALP (xULN): alkaline phosphatase expressed as times the upper limit of normal, AMA: anti-mitochondrial antibodies, Clinical FU: clinical follow-up after sample collection, UDCA: ursodeoxycholic acid

**Table 3:** Characteristics of PSC patients and controls

	Controls (n = 48)	PSC (all) (n = 47)	*p*-value^a^	PSC (early) (n = 24)	PSC (late) (n = 23)	*p*-value^b^
Sex, % male	58.3	59.6	> 0.9999	62.5	56.5	0.4158
Race, %Caucasian	100	98.0	0.4947	95.8	100	0.6085
Age at sample collection (yrs), median (IQR)	52.3 (44.1–62.1)	54.3 (32.8–62.1)	0.4847	47.0 (29.8–58.7)	57.4 (42.5–63.5)	0.1599
ALP (xULN), median (IQR)	0.59 (0.47–0.72)	1.03 (0.86–3.65)	< 0.0001	0.86 (0.74–0.95)	3.65 (2.96–5.36)	< 0.0001
Total bilirubin, median (IQR)	na	0.8 (0.5–1.8)	–	0.6 (0.5–0.9)	1.6 (0.8–3.4)	< 0.0001
Age at Dx (yrs), median (IQR)	na	42.4 (28.2–53.9)	–	36.2 (22.0–48.5)	46.1 (34.9–57.5)	0.0693
IBD type	na		–			0.3947
UC, n		36		18	18	
CD, n		2		2	0	
Ind. IBD, n		3		2	1	
None, n		6		2	4	
Disease duration (yrs), median (IQR)	na	6.0 (3.0–12.0)	–	5.0 (2.0–12.5)	6.0 (3.0–12.0)	0.8035
Clin. FU (yrs), median (IQR)	na	5.0 (3.0–6.0)	–	5.0 (4.0–6.8)	4.0 (1.0–6.0)	0.0699
UDCA treatment, (%)	na	70.2	–	69.6	77.3	0.7381

^**a**^*p*-value for control versus PSC (all) comparison

^**b**^*p*-value for PSC (early) versus PSC (late) comparison; ALP (xULN): alkaline phosphatase expressed as times the upper limit of normal, IBD: inflammatory bowel disease, UC: ulcerative colitis, CD: Crohn’s disease, Ind. IBD: Indeterminate IBD, Clin. FU: clinical follow-up after sample collection, UDCA: ursodeoxycholic acid

### Assay performance

**Fig. 1:**
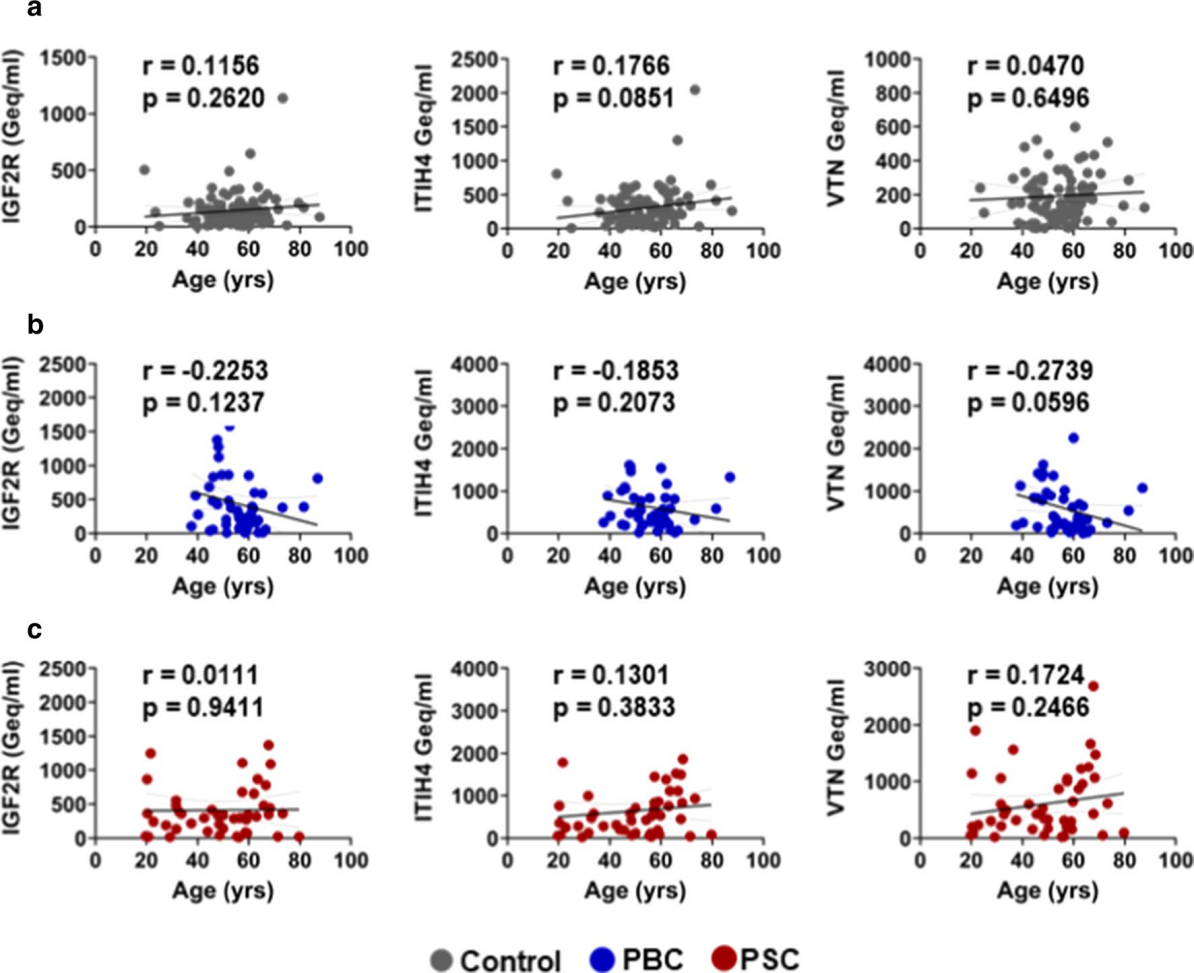
Lack of correlation between liver-specific cfDNA levels and participant age. Our study did not identify correlation between age and liver-specific DNA levels as measured at all 3 genes: IGF2R, ITIH4 and VTN in **a** Controls, **b** PBC patients or **c** PSC patients. Data presented as a plot of age in years versus cfDNA values expressed as genomic equivalents per ml (Geq/ml), with linear regression line and 95% confidence interval shown. Correlation was evaluated using the Pearson correlation coefficient (r)

**Fig. 2:**
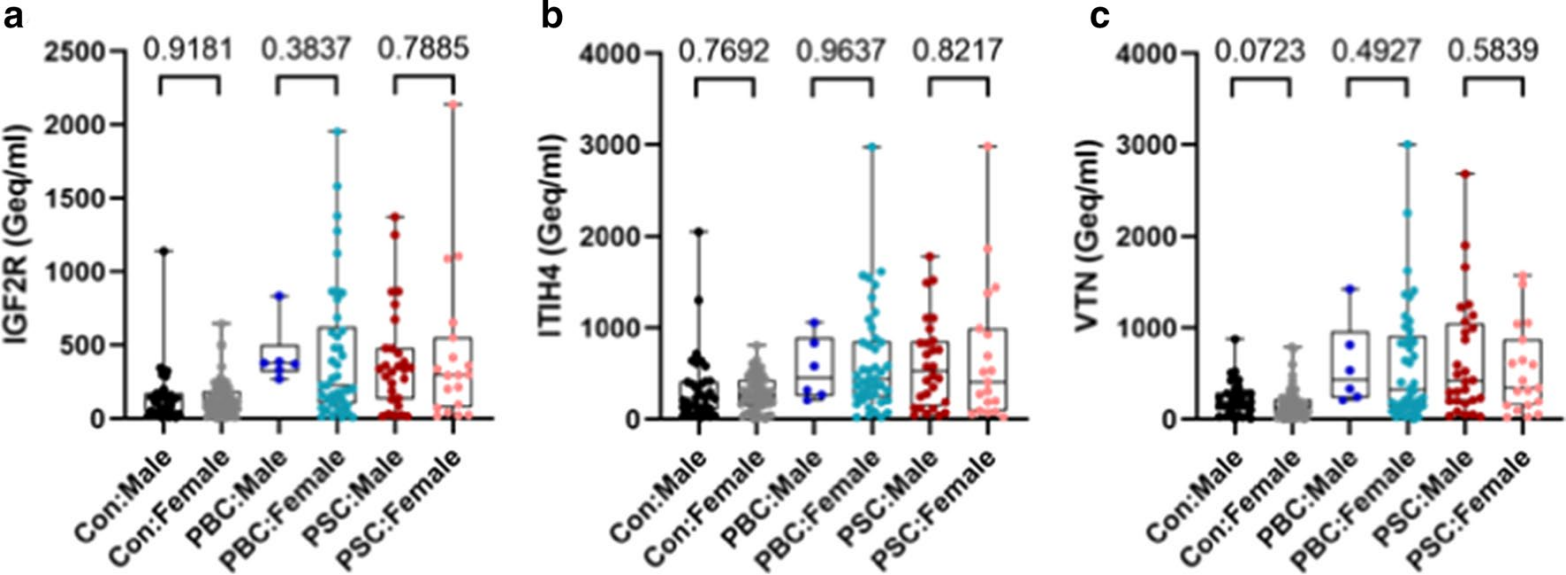
Lack of correlation between liver-specific cfDNA levels and participant sex. Our study did not identify correlation between sex and liver-specific DNA levels as measured at all 3 genes: IGF2R, ITIH4 and VTN in **a** Controls, **b** PBC patients or **c** PSC patients. cfDNA values expressed as genomic equivalents per ml (Geq/ml). *P*-values determined using the two-tailed Mann–Whitney test, exact *p*-values shown

We found that the multiplex amplicon-based method provides for very high read counts at each CpG site, with median counts in the range of 10,000 for CpGs in the VTN amplicon and over 30,000 for CpGs in the ITIH4 and IGF2R amplicons (Table 1). The ratios of unmethylated to methylated CpGs were relatively consistent in the CpG sites at IGF2R and VTN across the study population, with median values ranging from 0.030–0.041 to 0.045–0.064, respectively (Table 1). However, the unmethylated ratios of CpGs in ITIH4 were more variable, with one of the CpGs, ITIH4-1, being significantly higher than other evaluated CpGs with a median unmethylation ratio value of 0.280 (Table 1). This suggests either an assay-based artifact or that ITIH4-1 unmethylation may not truly be liver-specific, and thus, it was removed from the analysis. Liver-specific DNA concentrations in our controls seemed to be higher than those in the original report [12], possibly due to minor technical differences in the assay used. Consistent with the previous report [12], we did not detect an influence of age on liver-specific DNA concentration in controls (Fig. 1a). Likewise, age did not influence liver-specific DNA concentration in PBC (Fig. 1b) or PSC (Fig. 1c) patients. Finally, we found that sex did not influence liver-specific DNA levels as measured by all 3 genes: IGF2R (Fig. 2a), ITIH4 (Fig. 2b) and VTN (Fig. 2c).

### Liver-specific circulating cfDNA is increased in PBC and PSC patients compared to controls and in late-stage compared to early-stage disease

**Fig. 3:**
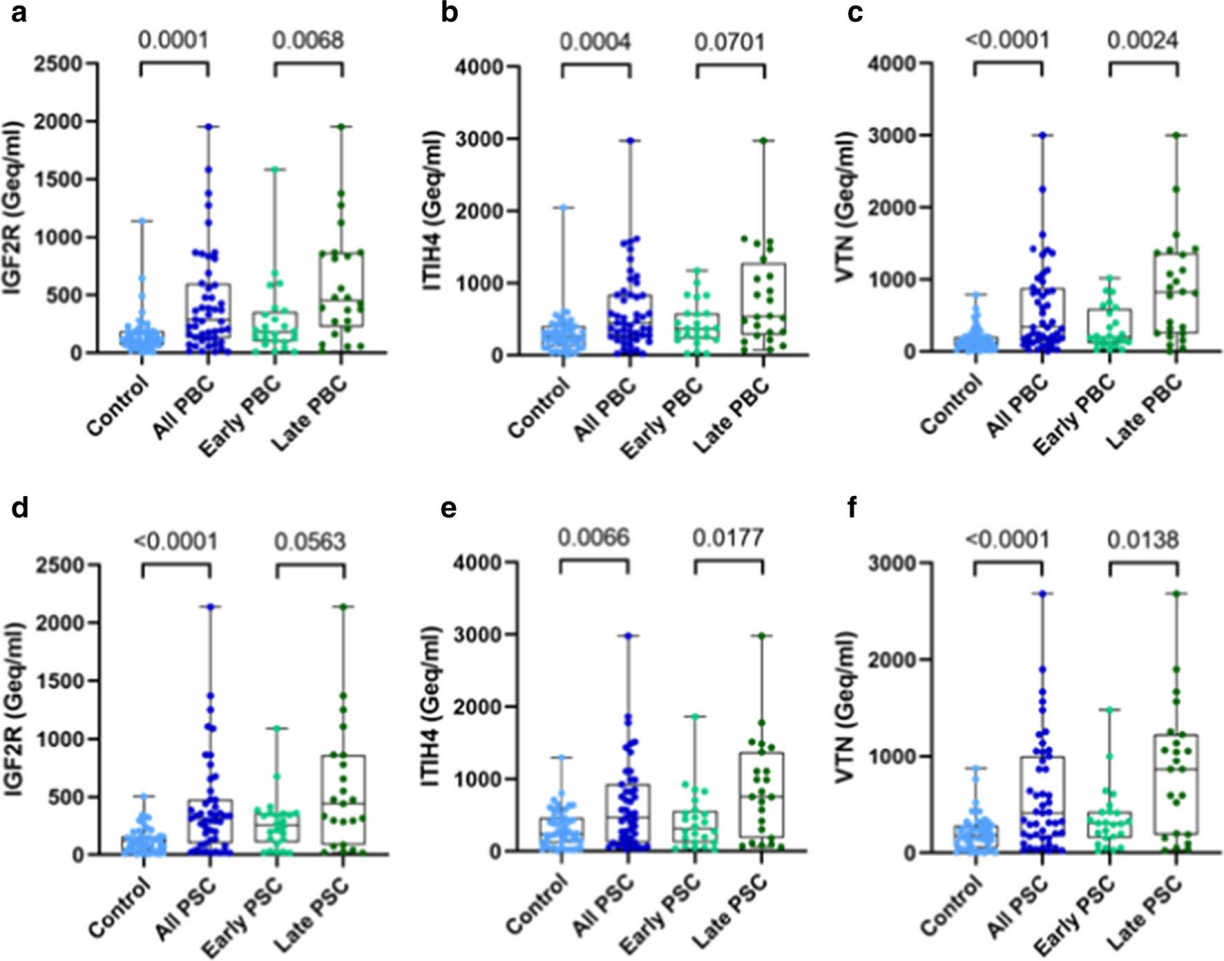
Liver-specific cfDNA levels increased in patients relative to controls and in late stage compared to early-stage disease. Comparisons of liver-specific cfDNA levels between patients and controls and between patients with early-stage and late-stage disease. PBC patients and controls **a** IGF2R locus, **b** ITIH4 locus and **c** VTN locus. PSC patients and controls **d** IGF2R locus, **e** ITIH4 locus and (F) VTN locus. cfDNA values expressed as genomic equivalents per ml (Geq/ml). *P*-values determined using the two-tailed Mann–Whitney test, exact *p*-values shown

The liver-specific circulating cfDNA (Geq/ml) values were used to make comparisons between patient and control groups and between patients with early- and late-stage disease. Results of these analyses are shown in Fig. 3. PBC patients demonstrated significantly increased liver-specific DNA compared to controls as measured by all 3 genes, IGF2R: (median (IQR)) 281.93 (126.24–597.61) Geq/ml versus 112.85 (66.71–184.19) Geq/ml, *p* = 0.0001 (Fig. 3a), ITIH4: 441.79 (256.40–837.79) Geq/ml versus 256.83 (122.99–399.69) Geq/ml, *p* = 0.0004 (Fig. 3b), and VTN: 334.73 (149.76–879.57) Geq/ml versus 133.13 (72.03–202.66), *p* < 0.0001 (Fig. 3c). These levels were also significantly increased in PBC patients with late-stage compared to early-stage disease when measured by IGF2R: (median (IQR)) 453.79 (221.47–862.67) Geq/ml versus 180.20 (95.97–355.83) Geq/ml, *p* = 0.0068 (Fig. 3a) and VTN: 822.01 (248.81–1359.55) Geq/ml versus 198.29 (114.34–595.37), *p* = 0.0024 Geq/ml (Fig. 3c). However, the increase when measured using ITIH4 was not statistically significant (median (IQR)) 539.26 (281.77–1273.28) Geq/ml versus 364.84 (225.98–581.50), *p* = 0.0701 (Fig. 3b).

PSC patients also demonstrated significantly increased liver specific DNA compared to controls as measured by all 3 genes, IGF2R: (median (IQR)) 317.46 (96.93–479.11) Geq/ml versus 122.10 (35.65–165.06) Geq/ml, *p* < 0.0001) (Fig. 3d), ITIH4 475.76 (124.81–929.76) Geq/ml versus 247.23 (117.87–470.36) Geq/ml, *p* = 0.0066) (Fig. 3e), and VTN: 415.12 (170.68–1000.30) Geq/ml versus 184.02 (53.83–285.65) Geq/ml, *p* < 0.0001) (Fig. 3f). These levels were also significantly increased in PSC patients with late-stage compared to early stage disease when measured by ITIH4 (median (IQR)): 759.85 (189.59–1377.80) Geq/ml versus 318.34 (121.44–566.00) Geq/ml, *p* = 0.0177 (Fig. 3e) and VTN: 867.24 (194.71–1227.01) Geq/ml versus 307.94 (159.98–427.80) Geq/ml, *p* = 0.0138 (Fig. 3f). However, the increase when measured using IGF2R was not quite statistically significant (median (IQR)) 443.17 (86.90–862.92) Geq/ml versus 257.40 (105.79–360.62), *p* = 0.0563 (Fig. 3d).

### Liver-specific circulating cfDNA levels are correlated with alkaline phosphatase levels in PBC and PSC patients but not in controls

**Fig. 4:**
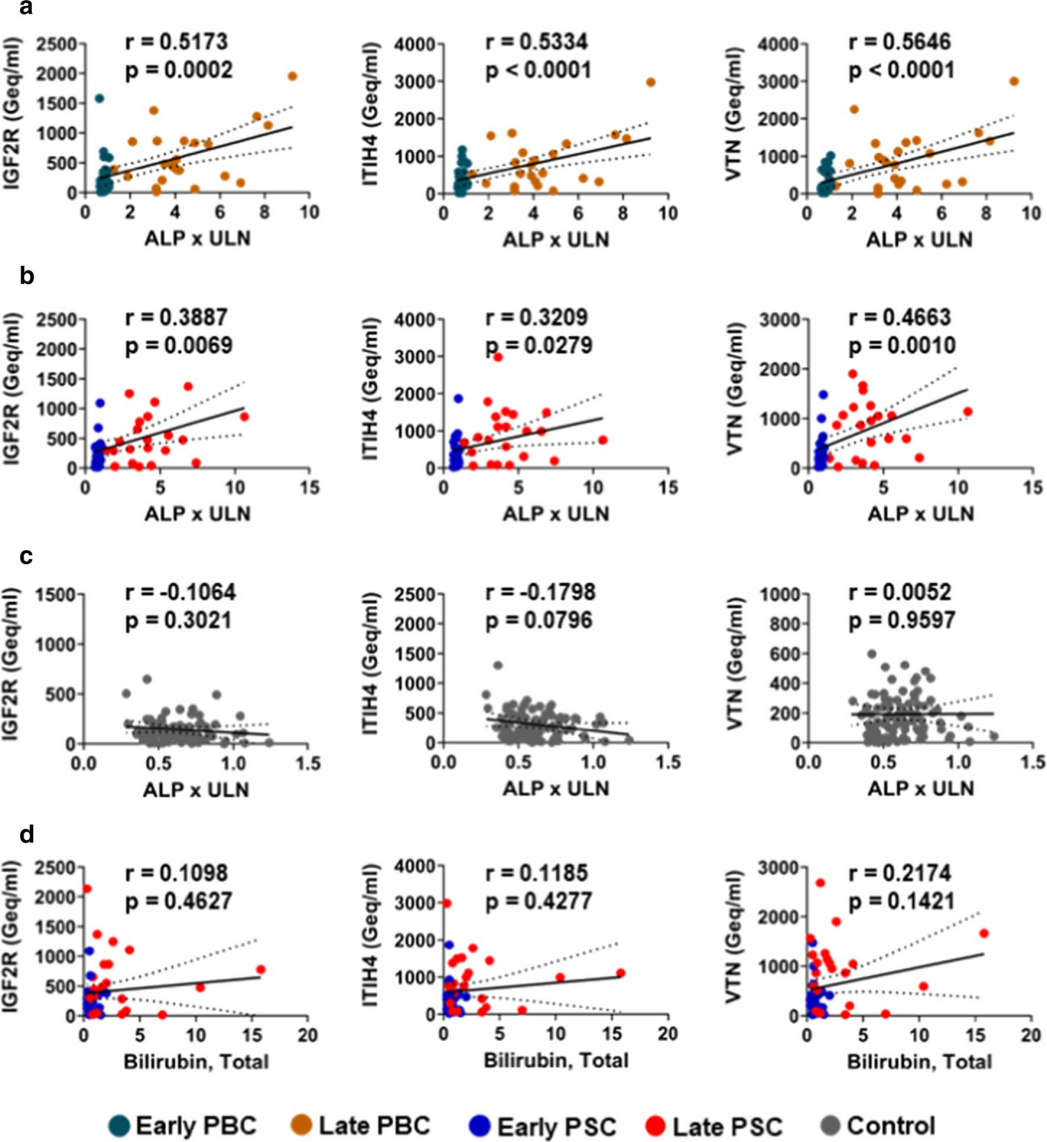
Correlation between liver-specific cfDNA levels and alkaline phosphatase in PBC and PSC patients. Alkaline phosphatase levels were correlated with liver-specific cfDNA as measured at all 3 genes: IGF2R, ITIH4 and VTN in **a** PBC patients and **b** PSC patients but not in **c** controls. **d** Total bilirubin levels were not correlated with liver-specific cfDNA levels in PSC patients. Data presented as a plot of alkaline phosphatase expressed as times the upper limit of normal (ALP x ULN) or Bilirubin, Total versus cfDNA values expressed as genomic equivalents per ml (Geq/ml), with linear regression line and 95% confidence interval shown. Correlation was evaluated using the Pearson correlation coefficient (r)

Liver function tests, particularly ALP, are often used to evaluate liver damage and disease severity in cholestatic liver diseases such as PBC and PSC [20, 21]. Thus, we evaluated the potential correlation between liver-specific circulating cfDNA and ALP (expressed as times the ULN) using the Pearson correlation coefficient. The results of these analyses are presented in Fig. 4 and show significant correlation between ALP and cfDNA levels as measured by all 3 genes in PBC (Fig. 4a) and PSC (Fig. 4b) but not in controls (Fig. 4c). We also had data available for Total bilirubin, another commonly used liver function test, in the PSC patients and found that those values did not correlate with liver-specific DNA levels as measured by any of the 3 genes (Fig. 4d).

## Discussion

Interrogation of organ-specific methylation patterns in circulating cfDNA is an emerging approach with great clinical potential, especially in the setting where traditional means of evaluation require invasive techniques such as biopsy. Such an approach would be particularly valuable for evaluating cholestatic liver diseases such as PBC and PSC as clinical guidelines do not recommend routine use of biopsy in these conditions due to risk of complications related to this invasive procedure. Here we demonstrate that liver-specific circulating cfDNA methylation patterns are increased in PBC and PSC patients relative to control groups and in late-stage compared to early-stage disease. As well, we demonstrate that the cfDNA levels correlate with ALP, a commonly used biochemical test to evaluate disease severity in PBC and PSC. Together, these findings suggest cfDNA assays may have potential clinical utility in cholestatic liver disease.

The bulk of research into the use of circulating cfDNA to evaluate disease has focused on noninvasive tumor evaluation [7], prenatal testing [22] and solid organ transplantation [23]; primarily exploiting differences in DNA sequence. Studies relying on organ-specific DNA methylation patterns have recently become more practical and are showing promise in a wide range of diseases including diabetes [24], cardiovascular disease [25] and neurodegenerative disorders [26]. Utility of cfDNA in the context of liver transplantation [27, 28] and other liver diseases including Hepatitis B [29], nonalcoholic fatty liver disease [30] and hepatocellular carcinoma [31] has been reported. However, to our knowledge, there has not been another study looking at the potential of cfDNA as a biomarker in PSC and PBC.

In our study we focus on an assay that interrogates CpGs at 3 genetic loci that were previously reported to be specifically unmethylated in the liver. For the genes IGF2R and ITIH4 the unmethylated state was described to be specific to hepatocytes, while VTN was unmethylated in both hepatocytes and cholangiocytes (i.e., biliary epithelial cells) [12]. However, methylation state of the CpGs in other major liver-resident cell-types such as Kupffer cells, liver sinusoid epithelial cells and hepatic stellate cells was not reported, and thus, a small proportion of the signal could be coming from these cells. Bile acid induced hepatocellular injury due to ongoing cholestasis has been long appreciated as a pathological feature of PBC and PSC [32] and the precise mechanisms of how this occurs are becoming more clear [33]. Thus, monitoring hepatocyte death as a proxy for ongoing disease activity is a valid approach, which our data supports. However, the use of cholangiocyte-specific epigenetic marks may prove more beneficial, particularly for PBC, in which cholangiocyte apoptosis plays a pivotal role in pathogenesis [34]. Indeed, discovery of cell-type specific epigenetic modifications in cholangiocytes and other liver-resident cells should be a priority for future studies seeking to utilize cfDNA to monitor cholestatic and other liver diseases.

While our study was designed to be able to detect the differences in cfDNA that we describe, there are limitations to our approach. First, we used stored plasma samples collected under variable conditions and thus, there could be the contribution of additional DNA from leukocytes that underwent cell death after sample collection in the cfDNA, potentially diluting the liver-specific signal. To avoid this, future studies should use samples that were purpose-collected using up-to-date methods and appropriate sampling tubes designed for collection of cfDNA. Second, we rely on amplicon-based next-generation sequencing, which is a time-consuming process. Future studies should focus on using emerging approaches such as digital droplet PCR [35], which once optimized can be performed quickly and reproducibly. Finally, there is significant inter-individual variability present in the data. Most notably, we find that some patients with early, and even late stage disease, have liver-specific cfDNA levels at the low end of what is observed in the controls. Whether this was due to variation in sample handling or is influenced by other factors such as ursodeoxycholic acid treatment remains to be determined. Larger studies, purpose-designed to evaluate such effects and the extent of intra-individual variability in cfDNA measurements over time will be needed to inaugurate clinical utility of cfDNA in PBC and PSC.

## Conclusions

In conclusion, cfDNA offers promise to become a non-invasive liquid-biopsy to evaluate liver-specific cell-death in patients with cholestatic and possibly other liver diseases. However, several challenges need to be overcome before this technology is ready for routine clinical use.

## Data Availability

The datasets used and/or analyzed during the current study are available from the corresponding author on reasonable request.
